# Critical Care Nursing in a Resource-Constrained Setting: A Qualitative Study of Critical Care Nurses’ Experiences Caring for Patients on Mechanical Ventilation

**DOI:** 10.1177/23779608231205691

**Published:** 2023-10-10

**Authors:** Paulus Mukuve, Vistolina Nuuyoma

**Affiliations:** School of Nursing and Public Health, Faculty of Health Sciences and Veterinary Medicine, 99404 University of Namibia, Windhoek, Namibia

**Keywords:** caring, critical care nurses, critical care unit, intensive care unit, mechanical ventilation, resource-constrained health systems

## Abstract

**Introduction:**

Managing a patient on mechanical ventilation is a vital aspect of clinical scope in intensive and critical care units. In addition, it is a highly technical, intricate, dynamic task requiring extensive knowledge and skills. Little is known about critical care nurses’ experiences caring for patients on mechanical ventilation in contexts where resources are constrained, creating an empirical gap in the available body of knowledge.

**Objective:**

This study explored critical care nurses’ experiences caring for patients on mechanical ventilators at an intermediate hospital in northeastern Namibia.

**Method:**

The study followed qualitative descriptive and explorative designs. The purposive sample included 13 critical care nurses who had cared for patients on mechanical ventilation for more than 6 months. Data were collected via individual unstructured interviews and analyzed using a reflexive thematic analysis approach.

**Results:**

Four themes and eight subthemes emerged. Varied personal feelings, such as feeling proud, competent, exhausted, traumatized, overwhelmed, and concerns for patients’ well-being were experienced by critical care nurses. Participants described learning from colleagues in the unit and expressed concerns about not having postbasic training in critical care nursing. Negative experiences included concerns about community members’ misconceptions about critical care units and mechanical ventilators, and challenges with resources, personnel, and admission procedures.

**Conclusion:**

Critical care nurses in resource-constrained settings have positive and negative experiences caring for patients on mechanical ventilators. The findings have implications for the development of support systems for critical care nurses, including induction programs, competence enhancement, psychological support, the development of guiding documents for admission, patient preparation and sensitization of community members. There is a need for this study to be replicated in other resource-constrained contexts where specialized critical care nurses are available.

## Introduction

Mechanical ventilation is a lifesaving intervention commonly used in caring for patients hospitalized in intensive and critical care units. It is a beneficial adjunct in patients with respiratory failure as it improves gas exchange and decreases the labor required for breathing (Shao et al., 2020). With invasive mechanical ventilation, an endotracheal tube channels mechanical breaths between the patient and the mechanical ventilator (Walter et al., 2018). Conversely, with noninvasive mechanical ventilation, an external interface, such as face masks and nasal cannulas, is used for ventilation (Popat & Jones, 2012).

Caring for critically ill patients entails nurses taking numerous decisions and maintaining acceptable standards of care in a highly technological environment (Acebedo-Urdiales et al., 2014). Managing a patient on mechanical ventilation is a complex and dynamic task that requires extensive knowledge and skills (Guilhermino et al., 2018) since breathing is a multifaceted process involving airways, lung parenchyma, muscles, the chest wall, peripheral nerves, and central nervous system. Therefore, patients on mechanical ventilation typically undergo multiorgan and multimodal monitoring (Roshdy, 2023).

Nursing activities in critical care units globally and in Namibia are influenced by a shortage of critical care nurses (Nakweenda et al., 2022). The Coronavirus disease of 2019 (COVID-19) pandemic recently placed significant pressure on acute care provision, burdening the healthcare system; especially in low- and middle-income countries. Nurses who are not trained and have no knowledge of critical care nursing have consequently had to step in as demand increases nursing staff gaps (Vincent et al., 2022). In upper-middle and high-income countries like Canada, COVID-19 also negatively affected the provision of critical care nursing due to the complex care patients required, placing increased physical and psychological demands on nurses (Gamble et al., 2022). Moreover, while managing a patient on mechanical ventilation is considered a vital aspect of the clinical scope in intensive and critical care units (Sweity et al., 2022), Saritas et al. (2019) reported many nurses who care for mechanically ventilated patients do not possess adequate knowledge of mechanical ventilation practices.

### Review of the Literature

Nurses’ experiences in critical care units differ from those in general wards due to the different scopes of care patients require. The use of life-sustaining technologies in critical care resulted in some nurses expressing insecurity over their competency with these technological tools. They also reported concerns that patients diagnosed with conditions with poor prognoses suffer longer as technologies prolong their lives, and nurses’ proficiency with technologies is seen as a challenge (Kongsuwan & Locsin, 2011). However, Cheng et al. (2021) determined that most nurses had a positive behavioral intention to care for patients undergoing mechanical ventilation, even during the COVID-19 pandemic. Nurses found caring for critically ill, nonsedated, mechanically ventilated patients a demanding yet rewarding task.

Nurses are responsive and attentive, and their closeness to patients enables interpretations of bodily and facial expressions (Laerkner et al., 2015). However, Limbu et al. (2019) reported that nurses expressed frustration in using old machines and equipment that did not function properly. As a result, they tended to focus more on machines than patients. In addition, nurses reported they required training to become competent in caring for patients on mechanical ventilation; they perceived themselves to possess good knowledge of how the machine operates but felt incompetent in using it. Furthermore, nurses in intensive and critical care units expect understanding and support from their management team. This support should be in the form of sensible rostering, patient allocation, and access to and time for counseling to promote their well-being. Nurses also claim their managers provide no resources, emotional support, or empathy to compensate for their overwhelm and fatigue (Jakimowicz et al., 2017).

As an additional challenge, Kyranou et al. (2022) reported barriers to communication between nurses and mechanically ventilated patients, attributed to mental, emotional, and physical conditions and medications’ influence on the patient's condition. Bäcklund et al. (2018) revealed nurses in intensive care units feel inspired, and patients recognize the importance of their presence. However, nurses felt frustrated by heavy workloads and had differing opinions on assessing patients’ pain and level of anxiety.

The provision of critical care is an intricate duty unique to regional, political, educational, socioeconomic, and cultural contexts (Losonczy et al., 2021; Macey et al., 2022). This is because personnel and physical resources vary based on socioeconomic status. Low- and middle-income countries reportedly have inadequate resources to meet healthcare system needs (Macey et al., 2022), affecting the provision of critical care nursing and nurses’ experiences of caring for patients on mechanical ventilation. This qualitative study explored critical care nurses’ experiences caring for patients on mechanical ventilators at an intermediate hospital in northeastern Namibia.

## Methods

### Study Design

The qualitative approach, which aligns with explorative and descriptive designs, was used. Explorative and descriptive qualitative designs are within the interpretative epistemological framework, helping researchers to interpret the meaning that individuals or communities assign to their experiences (Brink et al., 2018).

## Research Question

***“*** *What are critical care nurses’ experiences caring for patients on mechanical ventilators at an intermediate hospital in north-eastern Namibia?”*

### Study Setting

The critical care unit consists of 14 beds; four are used for mechanical ventilation, while 10 are for critical patients who do not require mechanical ventilation. In addition to the 14 beds, two beds are used for private noncritical patients who are cared for by the critical care nurses. This is the only critical care unit in the region, catering to three additional health districts and one neighboring region.

### Study Sample

The intermediate hospital employed 18 nurses in the critical care unit at the time of data collection. The study's sample comprised 13 purposively selected participants; the sample size was determined by data saturation. Data from new participants thus started repeating what previous participants stated during data collection. Additional evidence of saturation in this study is the absence of new codes or themes emerging during the analysis of the 13 transcripts, implying data were saturated (Saunders et al., 2018). Thirteen participants were considered adequate for this study; a systematic review by Hennink and Kaiser (2022) revealed saturation from empirical data is reached with sample sizes between nine and 17 interviews, with an average of 12 to 13 interviews. The inclusion criteria for participation in the current study were critical care nurses with more than 6 months’ experience caring for patients receiving mechanical ventilation. Critical care nurses who had cared for patients on mechanical ventilation for less than 6 months were excluded.

### Data Collection

Data collection occurred between July and August 2022. The researchers sought permission from the office of the hospital's medical superintendent before potential participants were approached in the critical care unit to seek their permission for participation in the study. An agreement was reached on the date and time of the interview, based on their availability. Ten individual interviews were conducted face-to-face in the hospital's common room. Three telephonic interviews were conducted with nurses scheduled for the interviews who traveled out of town but wanted to continue participation. All interviews were audiorecorded, and an interview guide was used. The interview guide was piloted with two participants prior to the main data collection phase, and necessary adjustments were made to guarantee high-quality research (Malmqvist et al., 2019).

Interviews were conducted by the first author, who is a registered and qualified critical care nurse and a student in the postgraduate nursing education program at the time this project was conducted. The second author, who holds a PhD in nursing education and is an experienced qualitative researcher, guided the data collection process as a project supervisor. Notes on the researchers’ reflections, participants’ nonverbal communication and body language were written in the field notes. The interviews ranged from 48 to 52 min, and audio recordings of the interviews were transcribed verbatim and shared with participants for member checking.

### Data Analysis

The two authors analysed data manually, following the reflexive thematic analysis approach outlined by Braun and Clarke (2006). This approach consists of six steps: data familiarization and writing familiarization notes; systematic data coding; generating initial themes from coded and collated data; developing and reviewing themes; refining, defining, and naming themes; and writing the report. Field notes were read together with transcripts during analysis, and this process assisted both authors in assigning meaning to the extracted data. The first author developed codes, subthemes, and themes from transcripts. The two authors then met face-to-face to reach a consensus on the final themes and subthemes for reporting.

### Data Quality Measure

The findings’ trustworthiness was ensured following Whittemore et al.'s (2001) primary criteria for validity in qualitative research, namely credibility, authenticity, criticality and integrity. Credibility was ensured by collecting data to saturation, audio recording all interviews, peer debriefing with other researchers who were not part of the study, and member checking with participants. Authenticity was ensured by verbatim transcribing all data from interviews while documenting all steps followed in the research process ensured integrity. In addition, findings are supported by participants’ quotes. Criticality criteria were achieved through the researchers’ engagement in critical thinking and critical appraisal of best practices in research methods before taking any steps in this project.

### Ethical Considerations

This study received ethical clearance from the departmental committee within the School of Nursing and Public Health at a public university, and the research unit of the Ministry of Health and Social Services. Participation was voluntary, and written informed consent was obtained from each participant prior to interviewing and audio recording them.

## Results

### Sample Characteristics

Ten participants were female, and three were male; their ages ranged from 23 to 46 years. One participant was categorized as a senior registered nurse, seven were registered nurses, and five were enrolled nurses. Their experiences working as critical care nurses ranged from 11 months to 10 years, yet none obtained specialized training in critical care nursing. This is not unusual; at the time of data collection, the intermediate hospital had only one registered nurse with formal specialized training in critical care nursing.

### Research Findings

Four themes and eight subthemes were extracted from reflexive thematic analysis. All themes and subthemes are indicated in Table 1.

**Table 1.** Summary of Findings.

Themes	Subthemes
Theme 1 Critical care nurses’ personal feelings	Feeling competent Concerned with patients’ well-being Exhausted, traumatized and overwhelmed by work
Theme 2 Training in the critical care unit	Learning from nursing colleagues No formal training and specialization in critical care nursing
Theme 3 Community members’ misconceptions of critical care units	
Theme 4 Challenges in the critical care unit	Staffing challenges Challenges with equipment Patients’ preparation and admission-related challenges

## Theme 1: Critical Care Nurses’ Personal Feelings

### Feeling Competent in Caring for Patients on Mechanical Ventilators

Participants gained experience working in the critical care unit and felt they were able to recognize problems in patients on ventilators, which they may manage themselves or inform doctors about. Participants sounded energetic and excited about caring for mechanically ventilated patients since they felt they had mastered all the required techniques to perform this task. In addition, they felt proud to care for critically ill patients on mechanical ventilation and described it as a rewarding job.

*Ventilated patients, I am able to assess and monitor the patient on those machines even when I am alone, I can do this and that, how to set up the machine, all that, I can do.* [Nurse 1]

### Concerned With Patients’ Well-Being

Participants were not prepared to face the pain and distress patients undergo while on mechanical ventilation. As a result, it made them worry about patients’ well-being and progress. They worried they would be unable to save patients and about patients’ likelihood of contracting infectious diseases such as COVID-19 and tuberculosis.

*Patients on mechanical ventilation are vulnerable, they can be admitted due to one problem and later develop another, for example it was difficult during COVID-19 time, some patients tested positive but were negative on admission. Not only that, but what if they get infected with a serious disease like tuberculosis.* [Nurse 10]

### Exhausted, Traumatized and Overwhelmed by Work

Participants indicated working with patients on mechanical ventilation was physically and emotionally exhausting. They reported anxiety, sleeplessness, emotional distress, and work felt overwhelming. In addition, the unit admits patients severely injured from accidents and some who are acutely ill, which were described as emotionally traumatizing for nurses. Observing many patients dying is traumatizing and ultimately affects work morale. These feelings were overwhelming, and working in critical care units was unattractive.

… *we go through disturbing experiences every day, people dying, injured casualties admitted, critically ill patients, even if patients are less, we get so busy trying to save them. It is most disturbing when you have done all you can, but patient pass on, we go through that every day.* [Nurse 3]

Moreover, nurses are often busy with administrative activities such as documentation, ordering medicine, stocktaking, and off-duty scheduling instead of offering direct nursing care to patients admitted to the critical care unit.

*Working here, I can say, its overwhelming, there is a lot of small tasks to do in-between caring of patients, you have to do ordering of drugs, food, do drugs control, do off-duty roster. I do not have a problem to care for patients but there's just those small tasks that can exhaust you.* [Nurse 6]

## Theme 2: Training in the Critical Care Unit

### Learning From Nursing Colleagues

Participants indicated they relied on colleagues with experience and those they found already working in the unit to show them how to operate ventilators and other equipment. They observed colleagues performing routine tasks and practiced under the supervision of an experienced colleague. In other words, they learned through trial and error until they mastered the skill.

*Not really training as such, but when I came to the ward, I found people who already knew the work done here, so they show me how to do certain things that I did not know. So, I would not really say it's a training in such because I'm just copying what people are doing.* [Nurse 11]

### No Formal Training and Specialization in Critical Care Nursing

Nurses’ placement in critical care units was based on their interests and the availability of vacant positions. None had formal training or postgraduate specialization in intensive and critical care nursing.

*… when I started working here, I can say it was a bit demanding because I didn't know how to monitor patients well, So I had to be trained almost like every week, not really like formal training but is like the doctor had to show me. What use to happen is that is when we had a patient on a ventilator, I will be shown how to monitor the patient, first it was tough and then later on with experiencing I improved.* [Nurse 11]

Working in critical care units without formal training is risky since nurses have no theoretical background. In most cases, participants depended on the guidance of doctors and one colleague who specialized in critical care nursing.

*…it is risky working here without formal training, because I will not actually know what to do when I encounter problems. Every time I have to call the doctor or the person who's trained if he's not there then it's a challenge I will not know what to do, it is not good to depend on other people all time.* [Nurse 7]

## Theme 3: Community Members’ Misconceptions of Critical Care Units

Members of the public often spread incorrect information about the critical care unit, especially after losing a relative. In some cases, community members believed ventilators suffocated patients who succumbed to their illnesses.

*I was once harassed by the family members of one patient who passed on here while under my care. Their allegations were that they left the patient breathing well, all of a sudden after I put him on a ventilator, the person died. They thought, I am God, or what?* [Nurse 9]

Moreover, there are also misconceptions that people admitted to a critical care unit do not recover since it only admits patients with poor prognoses. Some community members believed patients admitted for longer than three days in the unit die because nurses switch ventilators off as they become tired of caring for patients. They also claim some die due to negligence or poor management. It is believed that no patient should die in the critical care unit because it is well-equipped, and the care provided should be of high quality.

*I am a lifesaver and health care worker to ensure I do the best I can to the patient I am assigned to monitor. However, people do not understand, they believe I must not fail, or a person is guaranteed to be discharged healthy from ICU if they get admitted. They even think we switch off machines, neglect patients and let them die because we want less burden from our side, really.* [Nurse 10]

## Theme 4: Challenges in the Critical Care Unit

### Staffing Challenges

Participants expressed dissatisfaction with the nurse-to-patient ratio in the critical care unit. They reported few nurses are employed in the unit, leading to high workloads and no extra nurses to cater for emergencies in the unit. A staff shortage was also mentioned since some nursing staff allocated to the critical care unit were on leave.

*We do not have enough staff here because sometimes only two nurses on duty and when there's an emergency, eish … it is a disaster. Sometimes you are only two and there is five critical patients in the unit, and then they start gasping, we won't manage because we are only two.* [Nurse 11]

There is also sometimes a mismatch of nurses delegated to the unit in terms of their work experience and knowledge of critical care nursing.

*Sometimes, the way off-duty schedules are made, is really not helpful, you find junior nurses delegated together whole day, and really, patients suffer because they all lack knowledge on what to do, … they didn’t accumulate adequate experience or master the ventilator machines to work independently.* [Nurse 5]

### Challenges With Equipment

There is a critical shortage of equipment, pharmaceutical stock, and disposable items necessary for patient care, according to participants. In addition, available machines are frequently used past their intended lifespan, they are not regularly serviced or maintained and, therefore, are not functioning properly.

*Some machines are used for many years now, maybe passed expected age and some machine parts are difficult to replace, perhaps not regularly serviced because some are faulty.* [Nurse 8]

### Patients’ Preparation and Admission-Related Challenges

Participants had concerns about delaying decisions to transfer patients from general wards to the critical care unit. Transfers from the emergency department to the critical care unit are sometimes delayed, leading to poor prognoses and outcomes. Some patients arrived at the unit unprepared for admission, and time was spent on basic tasks, such as inserting intravenous lines, which were supposed to be done in the emergency department.

*What I have observed is that there are delays in deciding and action to transfer patients to critical care unit.* [Nurse 10]

Moreover, some patients admitted to the unit are chronically ill individuals and not classified as a priority. This was described as a major problem because there are limited beds, which should only be occupied by patients who need mechanical ventilation, which is unavailable in other units.

*…decision to admit patients in the unit depends on priority, which is based on the severity of illness, but now there is disorientation and a lot of confusions because even cold cases are admitted here, I don’t even know how it can be resolved.* [Nurse 13]

## Discussion

The study revealed critical care nurses’ varied personal feelings while caring for mechanically ventilated patients. These included feeling competent, proud, concerned for patients’ well-being, exhausted, traumatized, and overwhelmed by work. Another study focused on critical care nurses who cared for patients with a tracheostomy, and those participants were proud of the care they provided and described it as emotionally rewarding, especially after patients were discharged home (Akroute et al., 2022). In a study conducted in Spain, nurses in the critical care and emergency units reported feelings of anger, disgust, sympathy, compassion, empathy, embarrassment, guilt, shame, gratitude, and elevation (Jiménez-Herrera et al., 2020). Similarly, Toscano et al. (2022) reported emotional exhaustion, reduced personal accomplishment, and depersonalization as a sign of critical care nurses being overwhelmed by work. Critical care nurses in the current study reported feeling traumatized after witnessing many deaths and working with severely injured casualty patients, corresponding to other findings. Sezgin et al. (2022) reported nurses in their study felt devastated and faced an inability to help patients after observing a lot of deaths in their units. In addition, Gordon et al. (2021) revealed that critical care nurses experienced fear, worry, anxiety, stress, helplessness, and empathy, often attributed to being concerned about patients. Although their study did not focus on the experiences of critical care nurses caring for patients on mechanical ventilators, there were some similarities to the feelings critical care nurses experienced in the current study. The feeling of being competent reported in the current study is contrary to the findings of Limbu et al. (2019), who reported that critical care nurses self-reported that they are not competent in using ventilators.

Crawford et al. (2023) determined that critical care is underprioritized at a global level. As a result, there are inadequate practitioners trained in critical care, and for those who want to advance their education in this field, postgraduate fellowship training opportunities are unavailable. Moreover, in low- and middle-income countries, there is a greater shortage of trained individuals capable of caring for critically ill patients (Brotherton et al., 2021). In high-income countries like the United Kingdom (UK), new nurses in critical care units are taken through structured formal training programs, capacitating them in caring for critically ill patients and developing their self-confidence as professionals (Stewart, 2021). In Namibia, there is a postgraduate diploma in nursing science, which focuses on critical care as a specialization (UNAM, 2022). However, this does not solve the problem of a shortage of trained critical care nurses in public healthcare facilities, as most resign to join private hospitals after completing their training, leaving a significant gap in the public healthcare system. In the setting of the current study, only one nurse had completed a critical care postgraduate diploma at the time of data collection.

This study revealed that nurses rely on learning from colleagues who have adequate work experience in the unit. This concurs with Limbu et al.'s (2019) findings that other staff members demonstrated how to perform procedures in the unit to new critical care nurses. A lack of trained and specialized critical care nurses is worrisome as educational interventions are recognized as important tools for developing critical care capacity in resource-limited settings (Diaz et al., 2019). Besides that, having critical care nurse specialists, especially as heads of units, was associated with improved patient outcomes and fewer requiring mechanical ventilation (Fukuda et al., 2020). While the current study revealed some nurses were placed to work in a critical care unit soon after completing their undergraduate nursing training, Sezgin et al. (2022) claim newly qualified nurses’ placements to such units without receiving adequate training may lead to malpractice because they are inexperienced.

Critical care nurses in the current study mentioned that community members had misconceptions about critical care units. Some believe nurses switch off machines, while others claim patients suffocate due to the use of ventilators. Other misconceptions are that no patient should die in a critical care unit since it is well-equipped, while some believe patients do not recover since the unit only admits patients with poor prognoses. This information was spread by family members of patients who previously received mechanical ventilation. These findings are unique and not reported in previous research. Family members ultimately play a crucial role in the recovery process and should be actively involved in caring for patients on mechanical ventilation. Therefore, critical care nurses should build a good relationship with family members based on mutual trust and a common goal of providing the best support for the patient who is critically ill (Urner et al., 2018).

A recent study by Nakweenda et al. (2022) confirmed that the nurse-to-patient ratio in critical care units in Namibia is as high as 1:4. This exceeds the global nurse-patient ratio in critical and intensive care units, which should be 1:1 for ventilated patients and 1:2 for non-ventilated patients (Sharma & Rani, 2020), and is currently followed in high-income countries like Canada, the UK, Germany and the United States. Moreover, previous research indicated that nurses preferred to care for one patient on mechanical ventilation at a time, and they preferred more awake rather than sedated patients (Laerkner et al., 2015). High patient-to-nurse ratios are associated with problems such as rendering incomplete and delayed nursing care, neglected patients, and not administering medications and food on time (Nakweenda et al., 2022). The current study revealed staffing challenges related to high nurse-to-patient ratios, high workloads, and a mismatch in nurses’ work experience and knowledge of critical care nursing; some wanted to resign as a result. Alrabae et al. (2021) reported similar high workloads and physical and mental demands among nurses working in intensive care wards. Moreover, critical care nurses’ workload was significantly inflated by the high number of COVID-19-positive patients who required mechanical ventilation. This placed critical care nurses at risk of self-contamination and transmitting the infection to others.

Nurses frequently make mistakes when they are overworked, posing a danger to themselves, patients, and other members of the healthcare team (Lal et al., 2020). Moreover, there is an association between a high workload and psychological changes, such as feelings of hopelessness, unhappiness, anger, intolerance, introversion, tension, distraction, and self-insufficiency (Turan & Ançel, 2020). A high workload ultimately compromises patient safety and the delivery of quality care to critically ill patients while negatively impacting nurses’ well-being (Banda et al., 2022).

Critical care is among the most resource-demanding services in the health sector due to the highly technical environment needed to care for seriously ill patients (Falk, 2023). Unfortunately, the current study revealed challenges with equipment in the critical care unit. There is a critical shortage of equipment, pharmaceutical stock, and disposable items necessary when caring for patients on mechanical ventilation. Moreover, machines malfunction due to irregular maintenance, and some are used past their intended lifespan. In support, Malelelo-Ndou et al. (2019) reported on the nonavailability, insufficient amount, and poor quality of equipment in critical care units. Available equipment was often too old and not functioning properly, and there was a general shortage of medication, which was also the case in the current study. Brotherton et al. (2021) and Macey et al. (2022) found that low- and middle-income countries experienced equipment shortages as a major problem in providing critical care services. In high-income countries, healthcare systems are well-funded, and have structural support and needed resources (Papanicolas et al., 2018). A lack of resources is associated with substandard patient care, increased risk of infections and compromised patient safety (Malelelo-Ndou et al., 2019).

Admission delays to the intensive care unit where critical care is provided are associated with increased mortality (Oliveira et al., 2018). The participants shared concerns about patient admission delays to the critical care unit. Additionally, some patients are not prepared for critical care admission at the emergency departments, resulting in delays in the provision of care, leading to poor outcomes. Participants also narrated concerns about the limited number of beds and patients not being screened properly to identify who qualifies to be admitted to the critical care unit. As a result, the unit admits patients who are supposed to be in general wards, taking up beds of patients who need mechanical ventilation, which is only offered in critical care units. Malelelo-Ndou et al. (2019) also revealed challenges related to limited beds based on the number of patients who need admission for critical care. Literature confirmed decisions regarding admissions to critical and intensive care units are often guided by the admission and discharge policies (Bassford, 2017) available in high-income countries and some low- and middle-income settings. However, no intensive and critical care admission and discharge policy was available in the current research context.

### Strength and Limitations

One strength of this study was the use of the qualitative design, which allowed for unstructured individual interviews. This method allowed for open responses, leading to rich data on the real-world experiences of critical care nurses. This study's limitation lies in the fact that participants had no post-basic qualifications or specialized training in critical care nursing. Notably, they were considered critical care nurses at the intermediate hospital due to the nature of their work and the experience they had accumulated in the unit. It should also be noted that the research context had only one nurse formally trained and qualified as a critical care nurse through post-basic specialization. Thus, it is recommended that this study be replicated in contexts where there are more specialized critical care nurses, in Namibia and across other resource-constrained settings.

### Implications for Practice

The study's findings have implications for the development of induction programs, the enhancement of nurses’ competence through short courses and formal postbasic training, and the establishment of psychological support systems for critical care nurses. Moreover, other implications are the development of guidelines for patients’ admission and preparation for use in emergency departments and general units. Lastly, the findings may be used to help develop information pamphlets for community members to sensitize them about the role of critical care units and ventilator machines in the provision of healthcare services.

## Conclusion

Critical care nurses are primary carers for patients who are mechanically ventilated. While critical care nurses in this resource-constrained setting had no postbasic training in critical care nursing, they felt proud and competent in caring for patients on mechanical ventilation. However, they were also exhausted, traumatized, concerned about patients’ well-being, and overwhelmed by their work. There were concerns about community members’ misconceptions about critical care units and mechanical ventilators, and challenges with resources, personnel, and admission procedures. This study advances the available evidence of critical care nurses’ experiences caring for patients on mechanical ventilation in resource-constrained health systems. However, there is a need for this study to be replicated in other resource-constrained settings where more specialized critical care nurses are available.
